# COVID-19 and paediatric dentistry- traversing the challenges. A narrative review

**DOI:** 10.1016/j.amsu.2020.08.007

**Published:** 2020-08-21

**Authors:** Saleha Shah

**Affiliations:** a QMUL, UK; b Department of Paediatric Dentistry, Baqai Dental College, Baqai Medical University, Pakistan; c Alvi Dental Hospital, Pakistan

**Keywords:** Peadiatric dentistry, Infection control, Dental education, Dental practice management, COVID-19

## Abstract

The coronavirus disease (COVID-19) pandemic has become a major global public health emergency with a focus on preventing the spread of this virus for controlling this crisis. A dental setting is at a high risk of cross infection amid patients and dental practitioner's owing to the spread of infection via droplets suspended in the air by infected symptomatic or asymptomatic subjects. This review article informs about measures which reduce facility risk, manage symptomatic patients and protect personal health care and management with reference to paediatric dentistry.

## 1 Background

The COVID-19 pandemic has had a significant impact on dentistry. Recommendations have therefore been revised in response to this pandemic to respond to the unique changes for dental settings. They inform about the resumption of non-emergency dental care during COVD-19, facility and equipment, sterilization and disinfection protocols, provision of care to both COVID-19 positive and negative patients and recommendations on Paediatric Dentistry to minimize risk to patient and dental healthcare personnel (DHCP) [[1], [2], [3], [4]].

## 2 Risk of infection in a dental setting

Exposure to biological risk in a dental setting is a hazard to the patients, doctors, hygienists and assistants. When patients cough, sneeze or undergo procedures with ultrasonic, high speed instruments or air water syringe it results in aerosol, droplet, spatter, salivary secretions, debris or blood. This environmental spatter travels over a short distance, settles down quickly and contaminates the air, floor, operatory surface, medical supplies, devices, equipment, apparatus, dental health care professional and the patient. A salivary gland could be a major viral source enabling the transmission of COVID-19 by asymptomatic infections originating from infected saliva. Aerosols are liquid and solid particles (<50 μm diameter) suspended in air for protracted periods. Splatter is a mixture of air, water and/or solid substances (50 μm–7 mm in diameter). They may be contaminated with bacteria, virus and fungi hence pose a health risk to the DHCP. SMs offer around 80% filtration rate and good protection for elective dentistry in normal healthy patients. The COVID-19 measures around 120 nm (0.12 μm) and aerosol particle sizes range from 3 to 100 nm hence FFP3 respirator offers a filtration rate of 99% of all particles measuring up to 0.6 μm [5,6].

The routine aerosol generating procedures are not designed to offer protection against transmission of pathogens and the standard protective measures do not offer adequate effectiveness against patients generating aerosol in the incubation period, are unaware of the infection or conceal information regarding their infection [5,6]. Dental healthcare personnel (DHCP) are all paid and unpaid persons serving in dental healthcare settings with a potential for direct or indirect exposure to patients or infectious materials (body substance, contaminated medical supplies, devices, equipment, environmental surfaces, air). A DHCP is placed in the very high exposure risk category by OSHA via high potential for exposure to known or suspected viral sources for COVID-19 during specific dental procedures [[5], [6], [7]].

The risk of SARS-CoV-2 transmission via aerosols generated during dental procedures cannot be eliminated when practicing in the absence of Airborne Precautions (airborne infection isolation rooms or single-patient rooms, respiratory protection program, N95 respirators). It is vital to reduce the risk of infections in a dental setting by Infection control measures since unrecognized asymptomatic and pre-symptomatic infections have a likelihood of transmission in healthcare settings. SARS-CoV2 is sensitive to heat and ultra-violet rays. It is inactivated at 56 °C for 30 min and by lipid solvents ethanol, 75% ether, disinfectants containing peracetic acid, chloroform and chlorine but not by Chlorhexidine [7,8].

## 3 Controlling exposure to sources of occupational infection

This is essential in the reliable and effective protection of DHCP from exposure to pathogens. It includes engineering controls, administrative controls and personal protective equipment (PPE). The optimal way to prevent airborne transmission is via a combination of interventions from the hierarchy of controls including elimination (physical removal of hazard), substitution (replacing hazard), engineering controls, administrative controls and PPE (least effective control owing to a high level of worker involvement and dependence on proper fit and correct use). Source control entails coverage of the mouth and nose by a cloth face or a facemask to aid the reduction of risk of transmission of SARS CoV-2 from both symptomatic and asymptomatic people via respiratory secretions [7,8].

## 4 Implications for paediatric dental practice

Paediatric dental practice aims to maintain the well-being and safety of children during this Pandemic by redesigning, reconsidering and reflecting on the dental care practices and staying up to date with the current evidence based guidance and recommendations for child oral health care. Hence a risk assessment of the practice should be carried out to identify the measures required to minimize the risk of COVID-19 transmission.

### 4.1 Administrative controls and work practices

These work practices and policies reduce or prevent hazardous exposures. The practice should be cleaned thoroughly and clutter removed to facilitate frequent cleaning and disinfection. Toys, magazines and other frequently touched objects in the waiting area which cannot be cleaned or disinfected regularly are removed from the waiting area. Devise a protocol for receiving mails and deliveries. The number of dental setting/hospital/outpatient patients is limited and screened for respiratory illness prior to healthcare. Triage by telemedicine (telephones, video-call applications on cell phones, video monitoring or tablets) and manages patients suspected of COVID-19 without a face to face visit. If the signs and symptoms are present then the appointment is rescheduled. Clinical care is limited to one patient at a time. The essential personnel should only be allowed to enter a patient care area. Limit DHCP during a procedure to those essential for procedure support and patient care. This avoids multiple room entry and bundling. Entry of known or suspected COVID-19 should be restricted by the DHCP or allowed to enter with PPE. DHCP at higher risk for severe illness from COVID-19 (old age, chronic medical conditions or pregnant) should be excluded from caring for confirmed or suspected COVID-19 infection. DHCP recovered from COVID-19 (protective immunity) can care for COVID-19 patients [[9], [10], [11], [12], [13], [14], [15],[30], [31], [32], [33], [34]].[30], [31], [32], [33], [34]

DHCP should be monitored and managed with application of flexible sick leaves. HCP should monitor themselves regularly for fever and COVID-19 symptoms. They should stay at home if they feel unwell and cover their faces or leave the workplace if they start feeling unwell at work. Every HCP should be screened for fever and symptoms consistent with COVID-19 (Fever >100.0 °F, cough, shortness of breath, and sore throat) when their shift commences. Testing should also be done if temperature is < 100.0 °F or with other symptoms consistent with COVID-19. If COVID-19 is ruled out based on time and test and the DHCP has an alternate diagnosis (tested positive for influenza); the criteria for return to work should be based on that diagnosis. They should continue wearing a mask subsequent to returning to work, self-monitor for symptoms and reevaluate with a medical facility [24,[27], [28], [29]].

A DHCP should be trained on the use of N95 respirators (putting on, removal, limitations, maintenance, check seal, repair, replace) when caring for patients in aerosol generating procedures. They should undergo medical clearance and fit testing. A qualitative fit test is a pass/fail test relying on individual sensory detection whereas a quantitative fit test is a numerical measure of the effectiveness. Cohorting confirmed patients of COVID-19 in one area to confine their care prevents patient contact and minimizes the use of respirators. Just-in-time fit testing is the ability of a healthcare facility for larger scale evaluation, training and fit testing of HCP prior to receiving patients during a pandemic. An annual fit may be temporarily suspended during expected shortages [[9], [10], [11], [12], [13], [14], [15],[30], [31], [32], [33], [34]].

The set up should include clean or sterile accessible supplies and instruments for specific dental procedure only. Instruments should be stored in covered storage (drawers and cabinets) to maintain decontamination. They should be supplied when needed and disposed when the dental procedure concludes. Safety and quality assurance checks on radiographic equipment should be performed. AED should be tested as well. All the emergency drug kits should be checked for expiry. Ensure that the rechargeable items are fully charged and operational. Check the drinking water dispenser for staff use and recommission by manufacturer's instructions. The computer updates should be checked and installed. Minimally invasive/atraumatic restorative techniques (hand instruments) should be prioritized, aerosol-generating procedures via dental handpiece and air/water syringe should be avoided and ultrasonic scalers discontinued. When aerosol-generating procedures are necessary use four-handed dentistry to droplet spatter and aerosols may be minimized via a high evacuation suction and dental dam. Preprocedural mouth rinses (PMRs) with an antibacterial product (chlorhexidine gluconate, essential oils, povidone-iodine or cetylpyridinium chloride) may reduce the level of oral microorganisms in aerosols and spatter generated during dental procedures however (PPMR) do not have evidence regarding their clinical effectiveness to reduce SARS-CoV-2 viral loads or to prevent transmission [[9], [10], [11], [12], [13], [14], [15],[30], [31], [32], [33], [34]].

### 4.2 Engineering controls

Barriers (glass/plastic windows/curtains) in reception areas where patients report on arrival (intake desk, triage station, information booth, pharmacy and drop-off/pick-up windows are placed to reduce the risk of exposure between the potentially infectious patients and DHCP. Aerosol-generating procedures for patients confirmed or suspected of COVID-19 should take place in an airborne infection isolation room (AIIR). Air should be exhausted directly outside or filtered directly via a high-efficiency particulate air (HEPA) filter before recirculating. The expedient patient isolation room method involves high-ventilation-rate, negative pressure, inner isolation zone within a “clean” larger ventilated zone by a portable fan device with high-efficiency particulate air (HEPA) filtration. It increases effective air changes per hour of clean air to the room thereby reducing the risk to persons entering without respiratory protection. Twelve air changes per hour are recommended for a renovation or new construction. The ventilated headboard is a special inlet system to provide an improved air intake for a corresponding high-efficiency particulate air (HEPA) fan/filter unit Ventilation system with proper engineering controls (filtration, exchange rate) should be installed and maintained to provide movement of airflow in a direction from a clean (DHCP workstation or area) to a contaminated area (sick patient/clinical patient) (appropriate filtration, exchange rate) should be installed and maintained. Air supply in the receptionist area with return air louvers positioned in the waiting area achieves this effect. A heating, ventilation and air conditioning (HVAC) professional can increase the filtration efficiency to the highest level without deviation from designed airflow as well as increase the percentage of outdoor air supply. Demand controlled ventilation (temperature setpoint and/or occupancy controls) should be limited during occupied hours and up to 2 h post occupancy to ensure that ventilation remains unchanged. Bathroom exhaust fans should run continuously during work hours. A portable HEPA air filtration unit may be considered during and following an aerosol procedure. These units reduce particle count (droplets) and turn over time in the room rather than just relying on the building HVAC system capacity. A HEPA unit should be placed within the vicinity (chair) of a patient but not behind DHCP and the DHCP should not be positioned between the unit and the patient's mouth. The position of a unit ensures that the air is not pulled into or past the breathing zone of the DHCP. An upper-room ultraviolet germicidal irradiation (UVGI) is used as an adjunct to higher ventilation and air cleaning rates. Follow environmental cleaning and disinfection procedure with hospital-grade disinfectant [[9], [10], [11], [12], [13],[31], [32], [33], [34], [35], [36]].

### 4.3 Patient placement

Dental treatment should ideally be provided in an individual patient room when possible. In open floor plan dental facilities with open floor plans the spread of pathogens man be prevented by a distance of at least 6 feet between patient chair, easy-to-clean floor-to-ceiling barriers to enhance the effectiveness of a portable HEPA air filtration systems without interfering with the fire sprinkler systems and physical barriers between chairs for patients. The dental operatory should be parallel to the direction of airflow where feasible. In vestibule-type office layouts consider patient orientation by placing the head near the return air vents, away from pedestrian corridors, and towards the rear wall. The maximum number of patients who can receive care at the same time in the dental facility safely is determined by the layout of the facility, number of rooms and the time needed to clean and disinfect the operatories. It is advised that DHCP should wait at least 15 min after the conclusion of dental treatment and exit of the patient to commence the room cleaning and disinfection process. This allows droplets to sufficiently fall from the air after a dental procedure [3,24].

### 4.4 Personal protective equipment: respiratory protection (PPE)

Standard Precautions assume that each person is potentially infected or colonized with pathogens which may be transmitted in a healthcare setting. Standard Precautions entail a N95 (standard and surgical medical respirators) or facemask, eye protection (goggles, protective eye wear with solid waste shields, or a full face shield), and a gown or protective clothing during procedures which produce splash, spatter of blood or body fluid and known/suspected COVID-19 patients. A respirator is a PPE device worn on the face, covering least the nose and mouth to reduce the risk of inhaling hazardous airborne particles (dust and infectious agents), gases, or vapors. Respirators are certified by CDC/National Institute for Occupational Safety and Health (NIOSH). Surgical respirators are indicated for respiratory protection in airborne pathogens (tuberculosis, measles, varicella) and fluid hazard (high-velocity splashes, sprays, splatters of blood or body fluid) hence preferred over a facemask. An effective face seal of a respirator requires qualitative or quantitative measurement. The highest level of surgical mask should be used. A faceshieId is worn over a standard N95 when a surgical N95 is unavailable. N95 masks need to be conserved during the crisis period. Conventional capacity provides patient care sans any alteration in existing daily practices. Contingency capacity practices may be used provisionally during expected periods of N95 respirator shortages without significant impact on patient care [[9], [10], [11], [12], [13],[16], [17], [18], [19], [20], [21], [22], [23]].

Alternatives to N95 masks include filtering facepiece respirators N99, N100, P95, P99, P100, R95, R99, and R100, elastomeric half-mask (replace filter cartridges) and full facepiece air purifying respirators, powered air purifying respirators (loose fitting hoods or helmets) reusable (PAPRs). All of these provide equivalent or higher protection than N95 respirators when worn properly. Filtering facepieces with an exhalation valve are not used in a sterile surgical setting since the unfiltered exhaled air compromises a sterile field. N95 may be retained, reserved and used beyond the shelf life during shortage in the pandemic. They may be re-used by one HCP for multiple encounters with different patients but removed (doffing) after each encounter. The time in between re-use should not exceed the 72 h expected survival time for SARS-CoV2. N95 contaminated with gross blood, respiratory or nasal secretions and/or other bodily fluids should be discarded. Contamination may be reduced/prevented by wearing a facemask over it [[9], [10], [11], [12], [13],[16], [17], [18], [19], [20], [21], [22], [23]].

A facemask block respiratory secretions produced by the DHCP from contaminating other persons and surfaces (source control) when worn with instructions in symptomatic patients suspected of COVID-19 or other respiratory infection (fever, cough) but not N95. DHCP should wear a facemask at all times while they are in the dental setting as a part of universal source control. They should be instructed to not touch or adjust their mask or cloth face cover and perform hand hygiene immediately before and after. Cloth face covering should not substitute a respirator or facemask. DHCP whose job does not require PPE (clerical) may wear cloth face covering. DHCP (dentists, hygienists, assistants) may wear cloth face covering when not engaged in direct patient care and switch to a respirator or a surgical mask when PPE is required. DHCP when leaving the facility at the end of their shift should remove their respirator or surgical mask and wear cloth face covering. Self-contamination is prevented by changing or laundering a cloth face covering saturated with respiratory secretions, soiled, dampened or posing difficulty for breathing. Hand hygiene must be performed before and after touch/adjustment of face cloth or face mask. N95 and face mask should be used according to the type of activity. A DHCP at a distance greater than 6 feet from a symptomatic patient does not need a face mask or N95. Facemask may be used in a DHCP within 6 feet of an asymptomatic patient for provision of direct patient care. In other countries respirators for occupational use are approved according to country-specific standards. Manufacturers sans NIOSH approval should only include products approved by and received from China. If the remaining supply of N95 is absent consider HEPA with facemasks. Extended use of facemasks and respirators should is undertaken only when the facility is at contingency or crisis capacity and has implemented all likely applicable engineering and administrative controls [[9], [10], [11], [12], [13],[16], [23],^37^,^38^].

### 4.5 Hand hygiene

Hand hygiene is indicated after all patient contact, contact with infectious material and prior to wearing and removing PPE (gloves) to remove any possible pathogen transfer to bare hands. ABHR with 60–95% alcohol or washing hands with soap and water for at least 20 s is advisable. For visibly soiled hands use soap and water before ABHR. Hand hygiene supplies should be available for the DHCP in every care location [[9], [10], [11], [12], [13],[16], [17], [18], [19], [20], [21], [22], [23]].

Use eye protection (goggles, disposable face shield) and remove them prior to leaving the operatory. Reusable eye protection (goggles) must be cleaned and disinfected according to manufacturer's instructions. The disposable eye protection should be discarded after use. Personal eyeglasses and contact lenses are inadequate [[24], [25], [26], [27]].

### 4.6 Gloves

Clean and non-sterile gloves should be worn upon entering the patient care area. They should be removed and discarded followed by immediate hand hygiene. If they are torn or heavily contaminated they should be changed. They should be educated about the signs and symptoms and diagnoses of skin reactions associated with glove use [[24], [25], [26], [27]].

### 4.7 Gown

A clean isolation gown is worn upon entry; changed if soiled and removed to be discarded in a waste or linen container before leaving. Cloth gowns are laundered post use. Gown shortage should be prioritized for aerosol generating procedures, splashes and sprays where high-contact patient care activities favor transfer of pathogens to the hands and clothing of DHCP [[24], [25], [26], [27]].

### 4.8 Sequences recommended for donning and doffing PPE

Before entering a patient care area perform hand hygiene and don a clean protective clothing or gown that covers skin and personal clothing likely to be soiled by potentially infectious material like blood, saliva, or other materials. If a gown and protective clothing become soiled they should be changed. A surgical mask or respirator is worn and the mask ties are secured on the crown of the head (top tie) and base of the neck (bottom tie). If loops are present hook the mask around ears. Respirator straps should be placed on the crown of the head and base of the neck and checked for a user seal check each time. Eye protection should not include personal eyeglasses and contact lenses. Hand hygiene is followed by putting on clean non-sterile gloves. They should be changed when torn or heavily contaminated prior to entering the room [24].

After completion of dental care gloves are removed and the gown or protective clothes are discarded in a dedicated container for waste or linen. Disposable gowns are discarded after each use and protective clothes are laundered after each use. Exit the patient care area and perform hand hygiene, remove eye protection carefully by grabbing the strap and pulling upwards and away from head without touching the front of the eye protection. Clean and disinfect reusable eye protection prior to reuse by the manufacturer's reprocessing instructions but discard disposable eye protection after use. Remove and discard surgical mask or respirator without touching the front. For a surgical mask untie (or unhook from the ears) and pull it away from the face carefully without touching the front. For a respirator remove the bottom strap by touching the strap only and bring it over the head carefully. For the top strap; grasp it and bring it over the head to pull the respirator away from the face without touching the front. Finally perform hand hygiene to follow standard precautions [24].

## 5 Adherence to standard and transmission-based precautions

### 5.1 Before arrival

Appoint each group of patients one personnel from the dental clinic who can be reachable 24/7 in case of an emergency in order to asses and determine the need to be seen. When scheduling appointments (elective) provide instructions to the patient to call ahead of the visit and discuss the need to defer/reschedule their appointment if they experience fever or symptoms of COVID-19 on the day of their appointment/visit. Impart advice about their own cloth face covering, irrespective of their symptoms before entering the dental facility. Schedule an appointment for possible COVID-19 patients by triage to determine the need for appointment versus management at home. If the patient has to attend an appointment they must call beforehand to inform triage personnel about their symptoms of COVID-19 as well as follow appropriate preventive actions throughout the visit to contain the respiratory secretions. If a face cloth is difficult to tolerate hold a tissue instead). If a patient is arriving via transport by emergency medical services (EMS) allow the healthcare facility to prepare for receiving the patient [24].

### 5.2 Upon arrival and during the visit

Monitor and limit the points of entry to the dental facility as well as post visual alert posters, signs in appropriate language for instructions on respiratory hygiene, hand hygiene, cough etiquette, facemask or face cover. Provide 60–95% alcohol-based hand rub, tissues and no-touch receptacles for disposal at the entrances, waiting rooms, check-in, elevators and cafeterias. Minimize overlap in dental appointments and ask the patients and attending visitors to wear a face covering or mask prior to entry irrespective of symptoms of fever and COVID-19. Set up physical barriers (plastic or glass windows) to limit close contact between triage DHCP and potentially infectious patients. Establish an outdoor triage station to screen individuals prior to entering the facility. The triage DHCP must wear a respirator/mask, eye protection and gloves for taking vitals and assessing patients for care until COVID-19 is considered unlikely [24].

Prioritize triage of suspected symptomatic COVID-19 patients. DHCP should inquire about the presence of fever, symptoms of COVID-19, or contact with patients with possible COVID-19 from every patient at the time of patient check-in. COVID-19 symptomatic patient should be isolated in an examination room with door closed and waiting space separated by 6 feet or more with easy access to respiratory hygiene supplies. They should not be allowed to wait among other patients. If they opt to wait in a personal vehicle or outside the facility they may be contacted by mobile phone when their turn arrives. In an afebrile patient (temperature < 100.4 °F) and otherwise without symptoms consistent with COVID-19 may be provided dental care using appropriate engineering and administrative controls, work practices, and infection control considerations. The patient should be asked to wear a face cover at the completion of dental care prior to leaving the treatment area. All new fevers and symptoms consistent with COVID-19 should be monitored. Inadvertent treatment of a patient confirmed to have COVID-19 later may occur even when DHCP screen patients for respiratory infections. The patient should be therefore be requested to inform the dental clinic if they become symptomatic or are diagnosed with COVID-19 within 14 days following the dental appointment. In a patient with fever strongly associated with a dental diagnosis (pulpal and periapical dental pain and intraoral swelling) but no other symptoms consistent with COVID-19; care can be provided with appropriate protocols [24].

### 5.3 Additional strategies to minimize chances for exposure

This depends on factors like level of SARS-CoV-2 community transmission, number of COVID-19 being cared for at a facility, healthcare-associated transmission and anticipated PPE or staffing shortages. The potential for patient harm if care is deferred needs to be determined. It is better to modify or cancel in-person group healthcare activities and implement virtual methods or schedule smaller in-person group sessions with a face cover and a distance of 6 feet apart. Postpone all the dental elective procedures, surgeries and non-urgent outpatients. If a patient with a dental emergency is highly likely to cause harm by deferring treatment it is advisable to provide care without delay in facilities less heavily affected by COVID-19; provide care without delay in your facility as opposed to transferring them or provide care without delay while resuming regular care practices [[24], [25], [26]].

## 6 Facility considerations

### 6.1 Dental equipment considerations

The manufacturer's instructions should be reviewed for instructions for use (IFU) on dental equipment for office closure, period of non-use and reopening for all equipment and devices. It may require maintenance and/or repair after a non-use period. Test the quality of water for DUWL prior to dental care to ensure that the standards for safe drinking water are met (<500 CFU/mL). Assess the need to shock DUWL of any devices and products that deliver water used for dental procedures. The standard maintenance and monitoring of DUWL should be continued according to the IFUs of the dental operatory unit and the DUWL treatment products. In the presence of dampness or mold (musky smell), determine the source of water entry, clean it up and remediate. Indoor temperature and humidity must be maintained within recommended ranges [[30], [31], [32], [33]].

The autoclaves and instrument cleaning equipment should be cleaned routinely and maintained in accordance with the manufacturer's schedule. Sterilizers should be checked with a biological indicator and matching control after a period of non-use prior to reopening. Maintenance air compressor, vacuum and suction lines, radiography equipment, high-tech equipment, amalgam separators and other dental equipment according to manufacturer's instructions [[30], [31], [32], [33], [34]]. Semicritical items in contact with mucous membranes or non-intact skin have a lower risk of transmission. They are heat-tolerant hence sterilized by using heat. A semicritical heat-sensitive item is processed with high-level disinfection. Noncritical care items have the least risk of transmission via intact skin hence cleaned only but if an article is visibly soiled it is cleaned following disinfection with an EPA-registered hospital disinfectant. In case of visible contaminated with blood or OPIM use EPA-registered tubercocidal hospital disinfectant. Blood spill can be managed by an EPA-registered hospital disinfectant effective against HBV and HIV, EPA-registered hospital disinfectant with a tuberculocidal claim (intermediate-level disinfectant) or an EPA-registered sodium hypochlorite product. The central processing area should be divided into sections for receiving, cleaning, and decontamination; preparation and packaging; sterilization and storage [34].

The handpeice should be run to run to discharge water, air, or a combination for a minimum of 20–30 s after each patient to physically flush out material that may enter the turbine and air and waterlines Heat methods can sterilize dental hand pieces and other intraoral devices attached to air or waterlines. Manufacturer instructions should be followed for cleaning, lubrication, and sterilization should be followed to ensure their effectiveness and longevity of hand pieces. Handles or dental unit attachments of saliva ejectors, high-speed air evacuators, and air/water syringes should be covered with impervious barriers which are refreshed after each use. Visible contamination requires cleaning with an intermediate disinfectant prior to replacing the barriers [[30], [31], [32], [33], [34]].

Radiograph cross-contaminate equipment and environmental surfaces with blood or saliva hence use an aseptic technique, wear gloves when taking radiographs and handling contaminated film packets, wear additional PPE (mask, eyewear, gown) for blood or other body fluid spatter. Heat-tolerant intraoral radiograph accessories may be heat sterilized for semicritical items like film-holding and positioning devices before patient use Digital radiography sensors and other high-technology instruments (intraoral camera, electronic periodontal probe, occlusal analyzers, lasers) are semicritical devices which may be cleaned, heat-sterilized or high level disinfected [[30], [31], [32], [33], [34]].

Reprocess heat-sensitive critical and semi-critical instruments by using sterilant/high-level disinfectants or low temperature sterilization method (ethylene oxide). Single-use devices are usually heat-intolerant cannot be reliably cleaned. Syringe needles, prophylaxis cups and brushes, and plastic orthodontic brackets, prophylaxis angles, saliva ejectors, high-volume evacuator tips and air/water syringe tips, cotton rolls, gauze, irrigating syringes should be disposed after each use. Endodontic burs, files, broaches, diamond and carbide burs should be considered single use. Laboratory items (burs, polishing points, rag wheels, laboratory knives) should be heat-sterilized, disinfected or discarded. Heat-tolerant items used in the mouth (metal impression tray, face bow fork) should be heat-sterilized. Articulators, case pans and lathe should be cleaned and disinfected. Semicritical instruments needed for immediate use or use within a short time may be sterilized unwrapped on a tray or a container system. Critical instruments for immediate reuse can be sterilized unwrapped if the instruments are transported in a sterile covered container during removal from the sterilizer and transport to the point of use however critical items should not be stored unwrapped [[30], [31], [32], [33], [34]].

Personnel subject to occupational exposure should receive training for infection-control in combination with standard precautions, engineering, work practice, and administrative controls to reduce occupational exposures to blood to prevent transmission of HBV, HCV, and HIV. DHCP are at a significant risk for acquiring or transmitting hepatitis B, influenza, measles, mumps, rubella and varicella which are vaccine-preventable. They should be vaccinated for Hepatitis B vaccine however routine immunization for TB is not recommended. The vaccine for Hepatitis C vaccine is still unavailable and the risk of HIV transmission in dental settings is extremely low. HBsAg-positive persons should be counseled about HBV transmission prevention and for medical evaluation [39].

### 6.2 Environmental infection control

Environmental cleaning and disinfection procedures should be followed correctly and consistently. Water should be run through pipes and taps in surgeries, kitchen, bathrooms and showers. A liquid chemical sterilant/high-level disinfectant should not be used as a holding solution or an environmental surface disinfectant. After working on a patient without suspected or confirmed COVID-19; wait 15 min after completion of clinical care and exit of each patient to begin to clean and disinfect room surfaces of the dental operatory. This allows droplets to fall from the air after a dental procedure to perform sufficient disinfection. Entrance in the operatory is delayed until time elapsed allows air changes to remove potentially infectious particles. Cleaning and disinfection procedures include cleaning of frequently touched surfaces or objects and aerosol generating areas with cleaners and water followed by application of Environmental Protection Agency-registered, hospital-grade disinfectant based on contact times shown on the product's label. Alternative methods for disinfection which can be instituted include ultrasonic waves, high intensity UV radiation, and LED blue light however their efficacy against COVID-19 virus is unknown. Sanitizing tunnels are not recommended for use by CDC [[39], [40], [41], [42], [43]].

The purpose of laundry is to protect the worker from exposure to potentially infectious materials throughout the stages of collecting, management and arranging of contaminated materials via PPE, work practice, containing, labels, ergonomics and hazard communication. Hot water washing is recommended at 160 °F (71 °C) at least for a minimum of 25 min. Chlorine residual of 50–150 ppm is attained during the bleach cycle and chlorine bleach is activated at 135°F–145 °F (57.2°C-62.7 °C) water temperature. Rinse cycles add a mild acid (sour) to neutralize the alkalinity in water soap, or detergent. Dry cleaning is an alternative cleaning process utilizing organic solvents (perchloroethylene) for removal of soil from fabrics that may have been damaged in conventional laundering. Waste should be handled with PPE and wastewater treatment facilities include oxidation with hypochlorite (chlorine bleach) and peracetic acid and inactivation via UV irradiation. Puncture- and chemical-resistant/heavy duty utility gloves should be worn for instrument cleaning and decontamination procedures. PPE should be worn during cleaning when splashing or spraying is anticipated. Food service utensils should be managed in accordance with the infection control policy [[39], [40], [41], [42], [43]].

Extracted teeth are potentially infectious hence disposed in medical waste bins. Extracted teeth sent to a dental laboratory for shade or size comparisons should be cleaned, surface-disinfected with an EPA-registered hospital disinfectant with intermediate-level activity (tuberculocidal). Extracted teeth containing dental amalgam should not be placed in a medical waste container that uses incineration for final disposal since they may be given to recycling company. They may be returned to the patient on request hence standard maintenance does not apply. Dental prostheses, appliances, and items used in fabrication (impressions, occlusal rims, bite registrations) must be managed by optimum communication and coordination between the laboratory and dental practice with appropriate cleaning and disinfection with an EPA-registered hospital tuberculocidal disinfectant. Parenteral Medication should be administered with an aseptic technique. A single syringe should not be used for multiple patients even if the needle has been changed Perform surgical hand antisepsis by an antimicrobial soap and water, or soap and water followed by alcohol-based hand scrub prior to wearing sterile surgeon's gloves [[39], [40], [41], [42], [43]].

## 7 Risk assessment and work restrictions for DHCP with potential exposure to COVID-19

The close contact with vulnerable individuals in dental settings requires a conservative approach to monitoring and applying work restrictions to prevent transmission from potentially contagious DHCP to patients, other HCP/DHCP and visitors. The contact tracing of exposed DHCP and application of work restrictions depends upon the degree of SARS-CoV-2 community transmission (Minimal–no, moderate) and their resources. High-risk exposures involve exposure of DHCP eyes, nose, or mouth to material potentially containing SARS-CoV-2 particularly from aerosol-generating procedure (prolonged exposure for 15 min or more). Exposure not included as higher risk include body contact with the patient without PPE and hand hygiene and touching the eye, nose, or mouth with the same hands. Exposures can occur from a suspected case of COVID-19 or from a person under investigation (PUI).

A record should be maintained for a DHCP exposed to PUIs. If the test results are delayed over 72 h or the patient is COVID-19 positive then the work restrictions apply. A DHCP with prolonged close contact with a patient, visitor, or DHCP with confirmed COVID-19 should be excluded from work for 14 days after last exposure, self-monitored for fever or symptoms consistent with COVID-19 and contact their medical evaluation and testing facility if fever or symptoms consistent with COVID-19 develop [44].

DHCP with risk exposures other than high exposure risk do not require work restrictions. They should follow infection prevention and control practices by wearing a facemask at work, self-monitoring for fever or symptoms consistent with COVID-19, not reporting to work when ill and undergoing screening for fever or symptoms consistent with COVID-196 when their shift commences. A DHCP who develops fever or symptoms consistent with COVID-196 should immediately self-isolate and contact a medical evaluation and testing facility. DHCP with travel or community exposures should inform their health facility for guidance on need for work restrictions. For COVID-19 confirmed symptomatic individuals consider the exposure window to be 2 days before symptom onset through the time period. For COVID-19 confirmed asymptomatic individuals determining the infectious period can be challenging. They must be considered potentially infectious commencing 2 days post exposure until they fulfill the criteria for discontinuation of Transmission-Based Precautions. If the date of exposure is undetermined, use a starting point of 2 days prior to the positive test through the time period when the individual fulfills the criteria for discontinuation of Transmission-Based Precautions. When reopening a practice Post COVID-19 shutdown follow the dental practice reopening guidelines [44].

## 8 Recommendations for paediatric dentistry

### 8.1 Dental caries risk assessment

It is based on patient specific risk indicators including prior caries experience and longitudinal evaluation of caries progression. Longitudinal evaluation at each visit considers cavitation of white spot lesion and increased dimension. Progression in the existing white spot lesion is considered an increased risk status. Other caries risk factors include a high frequency of fermentable carbohydrate, maternal caries and socioeconomic status of the family. Surgical management of the enamel carious lesions is based on visual detection, shadowing under the enamel and radiographic enlargement of lesion. Active surveillance of caries monitors initial carious lesion progression instead of definitive treatment. Active surveillance strategies include preventive therapy with compliance and recall [45].

Children are considered at a low caries risk if they have no caries, no new lesion in 1 year, no white spot lesions and belong to a high socioeconomic status. They receive a clinical examination at twelve months and a diagnostic radiograph at twenty four months. Preventive therapy includes tooth brushing twice a day with a fluoride toothpaste twice a day and fissure sealants. Restorative therapy is not indicated [45].

Children considered at a medium risk of caries have or have had one or more lesions per year and belong to a middle socioeconomic status. They require a diagnostic examination at twelve months and a radiographic assessment between twelve to fourteen months. Preventive regimen includes tooth brushing with fluoride toothpaste, a six monthly application of topical fluoride and provision of fissure sealants. Restorative therapy entails active surveillance of carious white spot lesions and proximal enamel lesions. Progressive and cavitated carious lesions maybe managed by restorative therapy or by an aerosol free topical application of Silver Diamine Fluoride [45].

Children are considered to be at a high risk if they have or have had one or more proximal lesion, have more than two lesions per year, have white spot lesions or enamel defects, active caries in a mother or caregiver, wear appliances, have a high sugar consumption and belong to low socioecenonmic status. They require a clinical examination at an interval of three months, a radiograph at an interval of six months and dietary analysis. Preventive care includes tooth brushing with a fluoride toothpaste twice a day, systemic fluoride supplements, professional topical fluoride application every 3 months, fissure sealants and brushing with a high potency fluoride gel in a child over 6 years of age. Restorative care includes surveillance of white spot lesions, restoration of proximal caries and restoration of progressing and cavitated lesions or treatment with topical application of Silver Diamine Fluoride [45].

### 8.2 Early childhood caries (ECC)

Early childhood caries (ECC) is defined as one or more decayed (non-cavitated or cavitated lesions), missing (due to caries) or filled tooth surfaces in any primary tooth in a child under 6 years. Severe early childhood caries (SECC) is defined as any sign of smooth-surface caries in a child < 3 years of age, and from ages 3 through 5, one or more cavitated, missing (due to caries), or filled smooth surfaces in primary maxillary anterior teeth or a decayed, missing, or filled score of greater than or equal to four (age 3), < than or = to five (age 4), or < than or = to six (age 5) [46].

Caries disease process initiates as early as the first year in the life of a child. This highly prevalent global chronic disease is cost intensive and impacts the quality of life of a child and their parents. Cries management is child specific management of caries process via primary, secondary and tertiary prevention. ECC reduction approaches focus on inter-professional care to ensure access to oral health for infants/toddlers and raise awareness regarding the adverse sugar consumption effects [47].

Primary prevention encompasses prenatal health care, avoidance of night time bottle feed with sugary drinks or milk, restricting sugar intake and frequency for children younger than 24 months, avoiding frequent/nocturnal breast or bottle feeding after 1 year, exposure to dietary fluoridate (water, milk, salt), use of an age appropriate amount of fluoride toothpaste containing at least 1000 ppm fluoride for brushing at least twice a day, dental visit in the first year of life and regular applications of 5% fluoride. Secondary Prevention comprises of more frequent fluoride application with fissure sealing for arresting caries progression prior to cavity formation. Tertiary prevention combines non-invasive and invasive management for cavitated lesions. Topical application of Silver Diamine Fluoride arrest and prevent non-cavitated and progressive cavitated dentinal lesions in an aerosol free environment [47].

### 8.3 Fluoride

Fluoride is the cornerstone of caries prevention. It functions by arresting or inactivating carious lesions as a therapeutic agent in NRCT (non-restorative caries treatment). Addition of optimum fluoride level to community water supply (0.5–1.1 mg/L) helps reduce the caries prevalence safely and effectively. Hence this stable public health practice benefits all the residents irrespective of their level of education, oral hygiene practices, socioeconomic background, employment status or access to oral health care [48].

Brushing the teeth twice a day with a fluoridated toothpaste containing 1000 ppm F at least and with an age appropriate amount on a tooth brush reduces caries effectively. The recommended use of fluoride toothpaste in a child up to first two years is 1000 ppm F, twice a day with a grain sized amount of 0.125 g. For a child between two to six years of age the recommended use of Fluoride toothpaste is 1000 ppm F, twice a day with a pea sized amount of 0.25 g. In a child over six years of age the recommended use of fluoride toothpaste is 1450 ppm F, up to full length of brush amount of 0.5–1.0 g. Hence this is a convenient, widespread, inexpensive and culturally approved approach to caries prevention in a safe and effective manner. The amount of toothpaste used is important since children tend to swallow the toothpaste which poses a risk for fluorosis. The recommended use of fluoride toothpaste based on standard prevention in children less than 3 years is 1000–1500 ppm F. Enhanced prevention for children over 3 years includes 1350–1500 ppm F and for Children over ten years includes 2800 ppm F [48].

Professional topical fluoride varnish application containing 5% F (22,600 ppm F) or gel containing 1.23% F (12,300 ppm F) may reduce caries in children. In a child over 6 years of age and at a high risk of caries 0.5% fluoride gels and pastes are recommended. Fluoride supplements consisting of tablets/lozenges and drops may be considered in fluoridated water deficient area. Their intake should not exceed 0.07 mg/kg body weight daily and should be used with care to prevent oral topical effects. Non-cavitated and cavitated dentinal lesions can be arrested or prevented successfully by the use of aerosol free SDF 38% Silver diamine fluoride containing 5% fluoride (44,800 ppm F) [48].

### 8.4 Minimally invasive dentistry

The current paradigm shift focuses on minimally invasive management strategies which arrest caries by assessing the caries risk, early detection of caries, implementing prevention measures, promoting enamel and dentine remineralization, instituting minimally invasive surgical interventions and repairing restorations conservatively as opposed to replacement. These techniques work well in conjunction with fluoride exposure and good oral hygiene for both non-cavitated and cavitated lesions. They allow the reversal of demineralized lesions (non cavitated) thereby arresting naturally. Proximal non-cavitated lesions may be managed by a micro invasive Infiltration method. Cavitated lesions can be arrested by topical application of aerosol free 38% SDF (Silver Diamine Fluoride). Surgical intervention advocates a minimal cavity design, conservative caries removal from deep lesion and adhesive restorative material [49].

### 8.5 Erosion

It is an irreversible tooth structure loss arising by chemical dissolution via intrinsic sources (gastric acid) or extrinsic sources (diet) but not bacterial acid. The thin and less mineralized enamel of primary dentition renders it more susceptible to erosion. Bulimia erodes the lingual surface of upper incisors whereas Gastrooesophageal Reflux erodes the molars. Dietary acid can erode any surface but it is avoidable by cutting down acidic food and beverage exposure. Diagnosis necessitates the need for discerning the etiology if the diagnosis is by location and level of erosion. Management includes regular monitoring and the use of a fluoride containing toothpaste or mouthwash containing stannous fluoride, addressing the etiology addressed and delaying the restorative intervention for monitoring. Minimally invasive techniques may be applied for restoration of teeth which hurt. A medical referral is indicated for patients with dental erosion due to GERD and Bulimia [50].

### 8.6 Periodontal disease

Periodontal assessment for primary teeth includes clinical and radiographic evaluation of the gingiva, periodontium, alveolar bone levels and tooth mobility. Permanent dentition should be assessed subsequent to complete eruption. Triaging helps evaluate conditions which may have four combinations. They may have healthy gingiva with healthy bone, healthy gingiva with diseased bone (eg Hypophosphatasia), diseased gingiva with healthy bone (eg Herpetic gingivostomatitis) and diseased gingiva with diseases bone (Neutrophil defects) [51].

Generalized gingivitis continuing over 2 weeks is viral in origin due to an underlying systematic cause. They require periodontal culturing to rule out anaerobic bacteria triggering an aggressive immune response (Papillon-Lefevre syndrome or neutorpenias). A medical referral with regular follow up assists in ruling out chronic idiopathic neutropenia, cyclical neutropenia and leukemia. Hypophosphatasia may be considered in a non-traumatic premature primary incisor loss before 4 years of age with a concomitant diagnosis of cementum pathology. Langerhan's cell histiocytosis may be deliberated in premature eruption of primary molars in the neonatal period. Diagnosis is confirmed upon presence of Birbeck granule in specimen for gingival biopsy from the molar region [51].

Effectiveness and compliance of medication for enhancing immune response in patients with systemic disease (GCF in cyclical neutropenia or insulin treatment in insulin-dependent diabetes) is ascertained by regular monitoring of gingival and periodontal health. Stem cell transplant may be carried out to improve immunity for improved periodontal health in children in Chronic Granulomatous Disease and Leukocuyte Adhesion Deficiency Disorder however it is very rare [51].

### 8.7 Molar incisor hypomineralization

MIH is a qualitative, enamel developmental defect which involves one or more posterior teeth with or without permanent anterior. They present as a demarcation, creamy/white to yellow to brown patches with or without post eruptive breakdown and sensitivity. It may range from mild to severe and impair tooth brushing. Primary molars with hypomineralization predispose the permanent dentition to a higher risk of MIH. Early diagnosis concomitant with prevention and restorative care prevents subsequent progressive breakdown, hypersensitivity and pulpal inflammation [51].

The adhesive restorations should include sound enamel since bonding for sealants and composites is compromised. Atypical amalgam restorations needing more retentive features may aggravate the tooth defect and result in a high failure. GIC temporization despite a high failure rate can be utilized. Aesthetics in mild incisors may be conservatively managed by combining etching, bleaching and sealing. Severe cases may be managed by microabrasion or composite veneers and a full coronal coverage for the molars. Restorations have a poor long term outcome in this dental anomaly. If one or more teeth are affected with severe MIH and pain consider extracting the first permanent molars prior to the eruption of second permanent molars (8–9 y). The occlusion will determine the need for an orthodontic alignment. Recall will prevent failure of restorations, recurrent caries and post eruptive breakdown. Sensitivity may be managed by topical fluoride varnish application and arginine desensitization paste however hypersensitivity may require local anaesthesia for restorative management [52].

### 8.8 Developing dentition

Malocclusion in the developing dentition needs recognition, risk factor identification (environmental, etiologic, premature primary tooth loss), diagnosis and optimum treatment. This contributes to a stable, functional and aesthetic occlusion in the permanent dentition. The developing dentition is evaluated clinically (palpation), by radiographs and functional analysis for habits, airway, tooth size and shape, anomalies, anterior and posterior crossbite, skeletal discrepancy, periodontal health for achieving a diagnosis. Breast feeding reduces non-nutritive habits which may otherwise require management appropriate for the child's development, comprehension, malocclusion and the ability to cope with treatment. Space maintainers prevent space loss due to premature primary tooth loss. Minor interceptive orthodontics can manage aesthetics in an increased overjet which predisposes the incisors to an increased risk of trauma [53].

### 8.9 Special care dentistry

In special care dentistry basic advice and dental intervention have a high impact on pain management and clinical outcomes. During the pandemic triaging, ranking, conceding and making challenging choices have become a daily actuality. Telecommunication can enhance communication and provide psychological counseling and advice for special needs patients however phobia, learning disabilities and attention deficit hyperactivity disorder (ADHD) do not tolerate any form of local anaesthesia require sedation and general anaesthesia which is currently suspended. They can benefit from alternative techniques (gradual exposure, behavioral management, hypnotherapy, professional cognitive behavioral therapy (CBT), desensitization methods, virtual goggles for distraction) in a more adjustable dental service. There is a need to balance and weight the clinical decisions and review service capacity and patient's safety regularly [54].

### 8.10 Dental emergency treatment

Commonly presenting acute oral conditions/problems need a modified and consistent management approach. Management of dental emergencies focuses on triage, relief from pain (analgesia) or infection (antimicrobial) and provision of care via remote consultation (videocall or telephone). Referral is indicated in unmanageable severe or uncontrolled symptoms with adequate documentation [55,56].

Dental triage of usually presenting dental conditions categorizes patients into three types. The first type requires advice and Self-help. They have mild -moderate symptoms which can be managed remotely by analgesics and antimicrobials. The second type requires urgent care. They have severe or uncontrolled symptoms which are unmanageable by a patient and require the patient to see a dentist in a designated urgent dental care center. The third type is emergency Care for emergency conditions which require immediate attention [55].

Acute Apical Abscess includes pain (localized to a single tooth); swelling of the gingiva, face or neck; fever, listlessness, lethargy and loss of appetite in children under 16 years of age. Management by self-help includes analgesics and antibiotics (swelling/systemic infection) with a recall after 48–72 h. Urgent care by extraction or drainage is needed for spreading infection without airway compromise or continuing or recurrent symptoms. Emergency care is indicated for spreading infection with an airway compromise or trismus [55].

Acute Periodontal Abscess/Perio-Endo lesions include pain and tenderness of gingival tissue, increased tooth mobility, fever and swollen/enlarged regional lymph nodes, presence of swelling on gingiva and gingival suppuration. Management by self-help includes analgesics and antibiotics (swelling/systemic infection) with a recall after 48–72 h. Urgent care is for spreading infection without airway compromise or continuing or recurrent symptoms. Emergency care is indicated for spreading infection with an airway compromise or trismus [55].

Acute Pericoronitis includes pain around a partially erupted tooth, swelling of gingiva around the erupting tooth, discomfort on swallowing, limited mouth opening, halitosis (unpleasant mouth odour), fever, nausea and fatigue. Advice and self help include analgesia, chlorhexidine mouthwash/gel or warm salt water mouthwash, gentle toothbrushing of the affected area with a small head toothbrush in combination with benzdyamine mouthwash, antibiotics (swelling/systemic infection) and recall after 48–72 h. Urgent care by extraction is for spreading infection without airway compromise or continuing or recurrent symptoms. Emergency care is indicated for spreading infection with an airway compromise and/or severe trismus [55].

Necrotizing Ulcerative Gingivitis/Periodontitis include pain (localized/generalized), swelling, gingival bleeding, halitosis, gingival ulceration, fever and malaise. Advice and self help include optimal analgesia, chlorhexidine or hydrogen peroxide mouthwash/gel, gentle toothbrushing of the affected area with a small head toothbrush in combination with benzdyamine mouthwash or spray and metronidazole as the antibiotic drug of choice [55].

Reversible pulpitis includes intermittent or stimuli associated toothache with tenderness to percussion. Advice and self help care recommend analgesia, repair of a missing filling with an emergency temporary repair kit from a pharmacy or online, avoidance of hot or cold food and to call back if the sypmtoms worsen [55].

Irreversible pulpitis includes sharp and spontaneous pain which lasts for several hours and keeps the patient awake and pain which is difficult to localize to a single tooth, it may be dull or throbbing and worsened by heat and alleviated by cold. Advice and self help recommend analgesia, cold water rinses and to call back if symptoms worsen. Urgent care is needed when the severe and uncomfortable pain prevents sleeping or eating. Management includes extraction at an urgent dental care centre [55].

Dentine hypersensitivity includes sharp, sudden or short duration pain and exposed root surface secondary to gingival recession. Advice and self help recommend application of desensitizing toothpaste to the affected area and avoidance of stimulus which include cold or acidic food and drinks [55].

Dry Socket includes pain which arises 24–48 h after extraction in the vicinity of site of extraction. The socket is tender with an unpleasant taste or odour and occasional swelling. Advice and self help include analgesis, warm salt water mouthwash and antibiotics in infection (spreading or systemic) or a patient who is immunocompromised. Urgent care is required for dressing if the pain is severe, uncontrollable and prevents sleeping or eating [55].

Post extraction haemorrhage entails bleeding which may be immediate, within a few hours secondary to inadequate initial hemostasis or within a few weeks due to possible infection. Advice and self help include no spitting or rinsing, gentle rinses with warm but not hot salt water mouthwash to remove the excess blood, placing a rolled up piece of cotton or gauze moistened with saline or water on the socket and firmly biting on it to maintain a solid and continuous pressure for 20 minutrs prior to checking for bleeding. The patient is advised to avoid smoking, exercising, drinking alcohol or disturbing the clot after the bleeding has stopped. Urgent care is required when the bleeding stops but is not brisk and persistent. Emergency care is recommended when the bleeding fails to stop, is brisk and persistent. The patient should be asked about anticoagulant medication (warfarin, clopidogrel, aspirin) [55].

Oral Ulceration include pain (lip and/or oral cavity), ulceration, inflammation, abnormal appearance and dehydration or listlessness or agitation if severe. Advice and self help for ulcers less than three weeks include chlorhexidine mouthwash under 7 y, analgesia or topical benzdyamine oromucosal spray, soft diet, keeping the dentures clean or use a repair kit for trauma from an adjacent tooth. If the ulcers are due to primary herpetic gingivostomatitis, herpes Zoster infection or in an immunocompromised patient consider antiviral agents (acyclovir or penciclovir) in the early stages. Urgent care is advised for ulcers persisting over three weeks. If the ulcers are due to an underlying medical condition then a medical practitioner should be consulted. Emergency care is for oral ulceration with severe dehydration [55].

Cracked, Fractured, Loose or Displaced Tooth Fragments lead to pain (generalized or localized), tenderness to bite, sensitivity to hot cold and sweet food, open cavity, missing section of a tooth or filling, sharp edge on the tooth, mobile tooth or fragment, mobility or loss of restoration, soft tissue trauma (tongue, lip, cheek), gingival inflammation or recurrent caries. Emergency care is indicated for inhalation of a piece of tooth, filling or restoration. Advice and self help for broken or fractured teeth and filling includes emergency temporart repair kit for sensitive teeth, analgesia and call back if pain persists. Prosthesis (crown, bridge or veneer) may be repaired by an emergency repair kit with analgesics for pain relief [55].

Ill fitting or loose dentures result in pain (general, localized), difficulty in speech and mastication. Advice and self help include analgesia, removal of denture and routine dental care when the services have resumed [55].

Trauma from a fractured or displaced orthodontic appliance causes pain and soft tissue injury. Emergency care is required if the airway is compromised or the patient inhales or ingests pieces of a fractured appliance such as brackets. However brackets pass the bowel without incident. For advice and help the patient maybe referred to the orthodontic guidelines (British Orthodontic Society) [55].

Avulsed, displaced or fractured teeth encompass a fracture of tooth or loss of structure, increased tooth mobility or several teeth mobile as a unit, displacement or elongation or an empty socket. Urgent care if a permanent tooth has been avulsed (knocked out) includes handling the tooth with care by the crown (white part) and avoid touching the root, washing the tooth briefly for ten seconds under cold running water if dirty, re-implant it in the socket and bite on it with a handkerchief gently to hold it in position. If it is not possible to re-implant the tooth, it may be transported in milk (not water) or in the mouth between molars and inside of the cheek. A permanent tooth which has moved out of its usual position to affect the bite should be referred to an urgent care center. A permanent tooth fracture involving pulp should also be referred to an urgent dental care center. A permanent tooth fracture of the enamel and dentine requires advice and self help for applying a desensitizing toothpaste, analgesia and soft diet. A primary tooth which has moved out of its position and interferes with the bite requires urgent care. If a primary tooth has displaced without affecting the bite advice and self help should include information about soft diet and analgesia. A primary tooth which has been avulsed (knocked out) requires advice and self help for analgesia and soft diet however it should not be re-implanted [55].

Dento-alveolar injuries include pain, bleeding, swelling, teeth/dentures which do not meet together, mobility, praesthesia, other problems related specifically to bone fractures (nose bleed, diplopia, visual loss). Emergency management is necessitated for severe bleeding which does not stop within 15–30 min, significant facial trauma, head injury or loss of consciousness and inhalation of a tooth or a tooth fragment. Advice and self help for cases which do not have an emergency includes cleaning the affected area by gentle rinsing with a mild antiseptic, removing foreign objects from the mouth, applying ice pack to the soft tissue injury and swelling, applying pressure with a finger to stop bleeding and analgesia. Antibiotics are not indicated for non-emergency situations [55].

Hence successful outcomes depend on an optimum advice and timely emergency dental care.

### 8.11 Analgesics for adults

Moderate dental pain can be managed in adults by Paracetamol, 2 × 500 mg tablets up to four times daily (4–6 hourly for 5 days or with Ibuprofen, 2 × 200 mg tablets up to four times a day (4–6 hourly) first after food. Severe dental pain can be managed by increasing the dose of ibuprofen to 3 × 200 mg tablets up to four times a day right after food or combining ibuprofen with paracetamol after food without exceeding the daily dose/frequency or by diclofenac, 1 × 50 mg tablet up to three times a day in combination with paracetamol. Maximum dose of drug in twenty four hours is 4 g paracetamol, 2.4 g ibuprofen and 150 mg diclofenac. Contraindications for diclofenac and a high dose of ibuprofen (more than 1.6 g daily) include moderate or severe asthma, renal impairment or hypersensitivity to aspirin. The regimen for adult patient requiring a proton pump inhibitor include Iansoprazole, 1 × 15 mg capsule daily for 5 days or Omerprazole, 1 × 20 mg capsule daily for 5 days [56].

### 8.12 Analgesic doses for children

Dental pain is managed in children by paracetamol suspension (120 mg/5 ml or 250 mg/5 ml) or tablet (500 mg). The age dependent dose can be given up to four times a day. The dose recommended for 6–12 month old is 120 mg, 2–3 years is 180 mg, 4–5 years is 240 mg, 6–7 years is 240–250 mg, 8–9 years is 360–375 mg, 10–11 years is 480–500 mg, 12–15 years in 480–750 mg and 16–17 years is 500 mg – 1 g. The alternative drug is Ibuprofen sugar free suspension (100 mg/5 ml) or tablet (200 mg). This age dependent drug is given up to three times a day. The recommended dose for 1–3 years is 100 mg, 4–6 years is 150 mg, 7–9 years is 200 mg and 10–11 years is 300 mg. The doses for 6–11 months is 50 mg and 12–17 years is 300–400 mg both of which are given four times a day as opposed to three. Combination of paracetamol and ibuprofen is not recommended without the advice of a medical practitioner [56].

### 8.13 Antimicrobials for adults

Dental infections in adults can be treated by antimicrobials which include Amoxicillin, 1 × 500 mg capsule, 3 times a day or Phenoxymethylpenicillin, 2 × 250 mg tablets, 4 times a day or metronidazole 1 × 400 mg tablet, three times a day. The dose of amoxicillin or phenoxymethylpenicillin may be doubled in severe infections (extra oral swelling, eye closing or trismus) [56].

### 8.14 Antimicrobials for children

Dental infections in children can be managed by amoxicillin, phenoxymethylpenicillin or metronidazole. Amoxicillin is administered as a sugar free oral suspension (125 mg/5 ml or 250 mg/5 ml) or capsule (250 mg). The age dependent dose can be given three times a day. The dose for 6–11 months is 125 mg, 1–4 years is 250 mg, 5–11 years is 500 mg and 12–17 years is 500 mg. The dose of amoxicillin for severe dental infections in children from 6 months to 11 years the may be increased up to 30 mg/kg (max 1 g) for 3 times a day. For severe infection in children between 12 and 17 years the dose of amoxicillin maybe doubled. Phenoxymethylpenicillin is available as a sugar free oral solution (125 mg/5 ml or 250mg/5 ml) or tablets (250 mg). The age dependent dose can be given up to 4 times a day. The dose for 6–11 months is 62.5 mg, 1–5 years is 125 mg, 6–11 years is 250 mg and 12–17 years is 500 mg. For severe infections in children up to 11 years the dose of phenoxymethylpenicillin can be increased up to 12.5 gm/kg for four times a day. For severe infections in children aged 12–17 years the dose maybe increased up to 1 g for four times a day. Metronidazole is available as an oral suspension (200 mg/5 ml) or a tablet (200 mg). The dose dependent medicine can be administered up to three times a day unless indicated otherwise. The dose for 1–2 years is 50 mg, 7–9 years is 100 mg and 10–17 years is 200 mg. The dose for 3–6 years is 100 mg given twice a day as opposed to thrice a day [56].

### 8.15 First line of antimicrobials for dental infections

Acute apical abscess, Acute periodontal abscess/perioendo lesions are managed by a 5 day course of amoxicillin, phenoxymethyl penicillin or metronidazole whereas Acute pericoronitis, Necrotizing ulcerative gingivitis/periodontitis can be managed by a 3 day course of metronidazole or amoxicillin [56].

### 8.16 Contraindications and cautions

It is necessary to check the patient's current use of analgesics before advising or prescribing analgesics. Paracetamol in many over the counter preparations should be identified in all medications which have been ingested. An overdose is dangerous because it may cause fatal hepatic damage that is sometimes not apparent for 4–6 days. Refer a patient for an emergency assessment if they ingest a therapeutic excess of more than the recommended daily dose [8 × 500 mg tablets for adults] and more than or equal to 75 mg/kg in any 24-h period. Paracetamol is the analgesic of choice for women who are breastfeeding. For a pre-term, or low birthweight infant seek advice from a GP. Absorption, distribution, metabolism, or excretion of paracetamol may be affected by an underlying medical condition. Paracetamol is a suitable analgesic option in most people with liver disease but dose reduction might be required for some patients with moderate or severe acute hepatitis. For people taking anticoagulants paracetamol is considered safer than aspirin or NSAIDs because it does not affect platelets or cause gastric bleeding. Patients should have their usual INR check planned and inform their clinician if they have been using paracetamol regularly. Use paracetamol and ibuprofen with caution in children (asthma). A GP should be contacted when uncertain about a patient's medical condition, current medication or suitable analgesia [56].

Use NSAIDs with caution and if absolutely necessary use the lowest effective dose for the shortest time possible. Patients already taking an NSAID (prescribed or not) regularly for a non-dental condition should not take an additional NSAID to control dental pain. Ibuprofen should be prescribed with caution for patients taking low dose aspirin since the administration of additional NSAID may reduce the cardioprotective benefit of low dose aspirin and increases the risk of GI bleeds. In patients taking low dose aspirin, if an NSAID is necessary to control the pain, consider ibuprofen up to 1200 mg maximum daily with a PPI or contact the GMP for advice. Elderly patients at increased risk of cardiovascular, renal, and serious adverse effects including GI bleeding and perforation, which may be fatal should be prescribed ibuprofen with caution not exceeding 1200 mg ibuprofen per day with a PPI. Diclofenac is contraindicated. Monitoring blood pressure, renal function, and features of heart failure may be required 1–2 weeks after starting or increasing the dose of an NSAID. Avoid NSAID's in people with dehydration, due to risk of acute kidney injury. Chronic alcoholism and alcohol dependence increases the GI risk is increased with NSAIDs hence avoid NSAIDs if possible or prescribe with a PPI. Prescribe ibuprofen with caution to people with cerebrovascular disease, ischaemic heart disease, peripheral arterial disease, or risk factors for cardiovascular events like hypertension, hyperlipidaemia, diabetes mellitus and smoking. Prescribe it with caution in cardiac impairment or mild to moderate heart failure (NSAIDs may impair renal function) but not in severe heart failure. Prescribe up to 1200 mg per day as a first-line option (lower dose than the 4 × 400 mg per day regimen recommended in the BNF for dental pain). For higher doses liaise with the patient's GMP. Monitor blood pressure, renal function, and features of heart failure may be required 1–2 weeks after starting or increasing the dose of an NSAID. Liaise with the patient's GMP to discuss. If in doubt about the severity of the patient's heart failure or appropriate analgesics, consult with their GMP. Prescribe NSAIDs with caution to people with inflammatory bowel disease (NSAIDs may increase the risk of developing or cause exacerbations of ulcerative colitis or Crohn's disease). Prescribe NSAIDs with caution to people with mild to moderate hepatic impairment (do not prescribe in severe hepatic impairment). Dose reductions and monitoring of liver function may be necessary. Prescribe NSAIDS with caution to people with severe renal impairment and avoid if possible since sodium and water retention may occur leading to deterioration in renal function and, possibly renal failure. If the patient cannot avoid using an NSAID and has impaired renal function, monitor renal function 1–2 weeks after starting or increasing the dose of an NSAID. Avoid concomitant use of NSAIDs with anticoagulants (e.g. warfarin, dabigatran) if possible. All NSAIDs can cause GI irritation and reduce platelet aggregation, which can worsen any bleeding event. If concurrent use is necessary be aware of the potential risks of bleeding. Consider giving gastroprotection. Liaise with the patient's GMP if a PPI is required but is not currently prescribed. Prescribe NSAIDs with caution for patients with bleeding disorders (e.g. Haemophilia, von Willebrand disease and clotting factor deficiencies). Consult with the patient's GMP or haematologist [56].

## 9 Discussion

The role of dental professionals in preventing the transmission of COVID-19 is critically important since it has the most risk of spreading the virus than any profession in relation to COVID-19. Dentistry follows the principle of universal precautions for cross-infection control to safe guard the dental health care professionals and the patients. Hence the strict cross infection control measures and the awareness of infectious diseases transmission are leading to a better level of infection prevention control and better personal protective measures in a dental setting. Acute/chronic oral medicine issues are managed over the phone and medication regimens are continued as previously prescribed to avoid detrimental effects of sudden change in pharmacotherapy. Organized urgent dental care delivered by DHCP in appropriate PPE (gowns, gloves, FFP3 masks and eye protection) with high-volume aspiration and other measures to reduce/avoid the production of droplets, splatter and aerosols by dental drills and saliva. The profound impact of the SARS-CoV2 pandemic on dentistry necessitates that a paediatric dentist stays up to date with the current resources and evidence based guidance on dental care for children. The revised consensus guidelines highlight revised infection control protocols, management of suspected and possible cases of COVID-19 virus and risk based management of paediatric dental emergencies with medication or intervention. Patients treated for COVID-19 in ICU will require care since they are at a high risk of deterioration of oral health [57,58].

## 10 Conclusion

COVID-19 viral transmission concern necessitates the implementation of specific protocols to reduce the risk and spread of infection from patient to another person or medical tools and equipment. This narrative review article discusses and suggests the modification of patient management, clinical practice, introduction of devices and organizational practices during the COVID-19 and the way forward with reference to paediatric dentistry.

## Ethical approval

Ethical approval not required N/A.

## Sources of funding

Funding not needed N/A.

## Author contribution

Saleha Shah- Sole author.

## Research registration number

1. Name of the registry:
2. Unique Identifying number or registration ID:
3. Hyperlink to your specific registration (must be publicly accessible and will be checked):

## Guarantor

Saleha shah self guarantor.

## Consent

Consent not required N/A.

## Provenance and peer review

Not commissioned, externally peer reviewed.

## Declaration of competing interest

I do not have any financial or personal relationships with the other people or organizations which could inappropriately affect my work.
